# Experimental validation of *in silico* model‐predicted isocitrate dehydrogenase and phosphomannose isomerase from *D* *ehalococcoides mccartyi*

**DOI:** 10.1111/1751-7915.12315

**Published:** 2015-09-16

**Authors:** M. Ahsanul Islam, Anatoli Tchigvintsev, Veronica Yim, Alexei Savchenko, Alexander F. Yakunin, Radhakrishnan Mahadevan, Elizabeth A. Edwards

**Affiliations:** ^1^ Department of Chemical EngineeringMassachusetts Institute of TechnologyCambridgeMassachusettsUSA; ^2^ Department of Chemical Engineering and Applied ChemistryUniversity of TorontoTorontoONM5S 3E5Canada

## Abstract

Gene sequences annotated as proteins of unknown or non‐specific function and hypothetical proteins account for a large fraction of most genomes. In the strictly anaerobic and organohalide respiring *D* *ehalococcoides mccartyi*, this lack of annotation plagues almost half the genome. Using a combination of bioinformatics analyses and genome‐wide metabolic modelling, new or more specific annotations were proposed for about 80 of these poorly annotated genes in previous investigations of *D* *. mccartyi* metabolism. Herein, we report the experimental validation of the proposed reannotations for two such genes (KB1_0495 and KB1_0553) from *D* *. mccartyi* strains in the KB‐1 community. KB1_0495 or *Dm*IDH was originally annotated as an NAD ^+^‐dependent isocitrate dehydrogenase, but biochemical assays revealed its activity primarily with NADP ^+^ as a cofactor. KB1_0553, also denoted as *Dm*PMI, was originally annotated as a hypothetical protein/sugar isomerase domain protein. We previously proposed that it was a bifunctional phosphoglucose isomerase/phosphomannose isomerase, but only phosphomannose isomerase activity was identified and confirmed experimentally. Further bioinformatics analyses of these two protein sequences suggest their affiliation to potentially novel enzyme families within their respective larger enzyme super families.

## Introduction

As one of the smallest free‐living organisms, *Dehalococcoides mccartyi* are important for their ability to detoxify ubiquitous and stable groundwater pollutants such as chlorinated ethenes and benzenes into benign or less toxic compounds (Maymó‐Gatell *et al*., 1997; Adrian *et al*., 2000; 2007a; He *et al*., 2003; Löffler *et al*., 2012). Only these strictly anaerobic niche specialists are capable of harnessing energy for growth from the complete detoxification of known human carcinogens, trichloroethene (TCE) and vinyl chloride (VC) (EPA, 2011, Guha *et al*., 2012) to benign ethene. This energy‐conserving metabolic process, termed reductive dechlorination, is catalysed by reductive dehalogenases, the respiratory enzyme system of *D. mccartyi*. Reductive dechlorination, also known as organohalide respiration, is useful not only for the bioremediation of toxic chlorinated solvents, but also a key component to comprehend the recycling of naturally occurring organohalides (Gribble, 2010; 2012). As such, the fundamental understanding of *D. mccartyi* metabolism, including the genes and enzymes involved in metabolic processes, has wide‐ranging implications for the remediation of toxic compounds and global element cycling (Gribble, 2012). So far, numerous systems‐level studies on *D. mccartyi* metabolism, including the construction of a pan‐genome‐scale metabolic model (Ahsanul Islam *et al*., 2010), and various transcriptomic and proteomic analyses (Morris *et al*., 2006; 2007; Johnson *et al*., 2008; 2009; Lee *et al*., 2012), have begun to shed light on key metabolic processes and associated genes.

Although *D. mccartyi* metabolism is well studied, the activities of only a handful of metabolic genes have been experimentally validated. These include an Re‐citrate synthase (Marco‐Urrea *et al*., 2011) involved in the tricarboxylic acid (TCA)‐cycle, a bifunctional mannosylglycerate (MG) synthase/phosphatase with a potential role in osmotic stress adaptation (Empadinhas *et al*., 2004), 12 reductive dehalogenases involved in respiration and energy conservation (Magnuson *et al*., 1998; 2000; Krajmalnik‐Brown *et al*., 2004; Müller *et al*., 2004; Adrian *et al*., 2007b; Tang *et al*., 2013; Wang *et al*., 2014), and two hydrogenases (Jayachandran *et al*., 2004; Nijenhuis and Zinder, 2005) potentially involved in the electron transport chain of *D. mccartyi*. Genome sequences of several strains of these bacteria (Kube *et al*., 2005; Seshadri *et al*., 2005) revealed the presence of ∼ 50% hypothetical proteins and proteins with unknown or non‐specific functions in their genomes. The primary gene annotations, including the annotations for more than 80 metabolic genes, were reviewed, and in some instances corrected during the construction and manual curation of the genome‐wide *D. mccartyi* metabolic model (Ahsanul Islam *et al*., 2010). Also, a recent systems‐level study (Ahsanul Islam *et al*., 2014) on *D. mccartyi* transcriptomes provided additional confidence to some of the proposed gene reannotations used in the metabolic model and helped predict putative functions for five hypothetical proteins. One such hypothetical protein was proposed to be a putative bifunctional phosphoglucose isomerase (PGI; EC 5.3.1.8)/ phosphomannose isomerase (PMI; EC 5.3.1.9) from the gene‐expression analysis (Ahsanul Islam *et al*., 2014) and metabolic modelling studies on *D. mccartyi* (Ahsanul Islam *et al*., 2010). Another metabolic gene that was primarily annotated as a putative NAD^+^‐isocitrate dehydrogenase (IDH) (EC 1.1.1.41) was reannotated as a putative NADP^+^‐dependent IDH (EC 1.1.1.42) during the modelling study. However, these proposed reannotations were not supported by any experimental data.

In this study, we report the heterologous expression and biochemical characterization of the aforementioned putative IDH (KB1_0495) and PGI/PMI (KB1_0553) from *D. mccartyi* in KB‐1 (Hug, 2012; Ahsanul Islam *et al*., 2014). KB‐1 is a mixed enrichment culture containing several strains of *D. mccartyi* (Duhamel and Edwards, 2006; Hug *et al*., 2012). These two genes were selected because they produced soluble proteins when expressed in *Escherichia coli*. The orthologs of these genes in pure strains of *D. mccartyi* (DET0450, cbdbA408, DehaBAV_0427, DhcVS_392, DehalGT_0391, btf_415, dcmb_461, GY50_0375 and DET0509, cbdbA472, DehaBAV1_0485, DhcVS_0450, DehalGT_0448, btf_472, dcmb_518, GY50_0435) are 98–100% similar at the amino acid level (Markowitz *et al*., 2012). Although IDH is an important TCA‐cycle enzyme catalysing the formation of 2‐oxoglutarate and CO_2_ with NAD^+^ or NADP^+^ as a cofactor (Nelson and Cox, 2006; Madigan *et al*., 2010; Kanehisa *et al*., 2011), the physiological role of a bifunctional PGI/PMI in *D. mccartyi* is unclear. PGI, in general, plays a central role in sugar metabolism via glycolysis and gluconeogenesis in all life forms (Hansen *et al*., 2004a, 2004b; Nelson and Cox, 2006), whereas PMI helps to produce precursors for cell wall components, glycoproteins, glycolipids and storage polysaccharides (Hansen *et al*., 2004a; Quevillon *et al*., 2005; Rajesh *et al*., 2012). However, glycolysis is inactive in *D. mccartyi* (Kube *et al*., 2005; Seshadri *et al*., 2005; Ahsanul Islam *et al*., 2010), and cells of these bacteria also lack a typical bacterial cell wall (Löffler *et al*., 2012). The genes (KB1_0495 and KB1_0553) were, thus, heterologously expressed in *E. coli*, and the purified proteins were experimentally tested for activities with enzymatic assays to confirm or refute the proposed annotations. Further bioinformatics analyses of their sequences suggested their affiliation to potentially new enzyme families within their respective larger enzyme super families.

## Results and discussion

### Biochemical activities of KB1_0495 and KB1_0553

The heterologously expressed and purified protein from KB1_0495 was tested for the IDH activity using a standard assay (Experimental procedures) to measure the conversion of D‐isocitric acid to 2‐oxoglutarate and CO_2_ using NADP^+^ or NAD^+^ as a cofactor. The pH range and activity of the enzyme (Table 1) were measured using D‐isocitric acid as substrate. The enzyme showed IDH activity using both NADP^+^ and NAD^+^ as the cofactor, but the activity with NAD^+^ was 65 times lower than with NADP^+^ (Table 1). This finding confirmed the annotation of KB1_0495 as an NADP^+^‐dependent isocitrate dehydrogenase (*Dm*IDH) proposed by the previous metabolic modelling study (Ahsanul Islam *et al*., 2010). The kinetic parameters for *Dm*IDH were also estimated using both NADP^+^ and NAD^+^ as cofactors (Table 1), and the values obtained for isocitrate and both cofactors are quite comparable with similar bacterial and archaeal enzymes in the BRENDA database (Chang *et al*., 2009). The higher activity and efficiency of *Dm*IDH with NADP^+^ are probably linked to its physiological roles in *D. mccartyi*. The incomplete TCA‐cycle of these bacteria is mainly used for anabolism (Tang *et al*., 2009; Marco‐Urrea *et al*., 2011). Hence, *Dm*IDH plays a crucial role in *D. mccartyi* metabolism by producing the critical biosynthetic precursor 2‐oxoglutarate and recycling essential cellular redox currency, NADPH. In fact, most bacteria possess NADP^+^‐dependent IDHs (Chen and Gadal, 1990; Muro‐Pastor and Florencio, 1994), and microbes having an incomplete TCA‐cycle use IDH to generate precursors (2‐oxoglutarate) and reducing power (NADPH) for anabolic biosynthetic pathways (Dean and Golding, 1997; Steen *et al*., 1998). However, NAD^+^ may also work as a cofactor for *Dm*IDH *in vivo* because the cellular concentration of NAD^+^ is ∼ 9 times higher than that of NADP^+^ in a bacterial cell (Wimpenny and Firth, 1972; Andersen and von Meyenburg, 1977).

**Table 1:** Kinetic Parameters of *Dm*IDH and *Dm*PMI from *D* *. mccartyi* ^a^

Enzyme tested (varying substrate)	*V*_max_ (μmoles·min^−1^·mg^−1^)	*K*_m_ (mM)	*k*_cat_ (s^−1^)	*k*_cat_/*K*_m_ (mM^−1^·s^−1^)
*Dm*IDH (isocitrate)^b^	21.3 ± 2	0.11 ± 0.01	15 ± 1	139.6 ± 1
*Dm*IDH (NADP^+^)	20.8 ± 2	0.027 ± 0.02	14.6 ± 1	540 ± 1
*Dm*IDH (NAD^+^)	0.32 ± 0.05	0.12 ± 0.04	0.22 ± 0.03	2 ± 1
*Dm*PMI (M6P)	1.19 ± 0.2	0.89 ± 0.09	0.84 ± 0.1	0.96 ± 0.3

**a.** Reaction conditions were as described in Experimental procedures. **b.** Kinetic parameters were determined using NADP^+^ as cofactor (0.3 mM).

**Figure 1:**
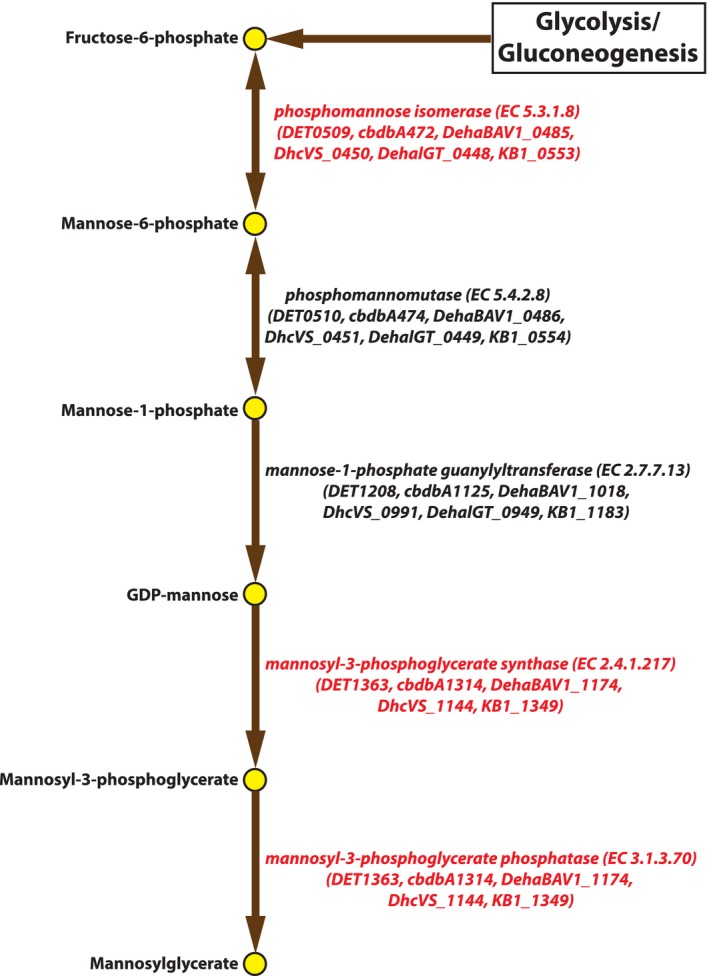
Mannosylglycerate (MG) biosynthesis pathway in *D* *. mccartyi*. Proposed genes and enzymes involved in the compatible solute, MG biosynthesis pathway in *D* *. mccartyi* are shown. Gene locus names of homologous genes in different *D* *. mccartyi* genomes encoding the enzymes in each step are shown in parenthesis. Biochemically characterized enzymes are highlighted with the red font colour. Phosphomannose isomerase (EC 5.3.1.8) was characterized in this work (KB1_0553, *Dm*PMI), and the bifunctional mannosyl‐3‐phosphoglycerate synthase (EC 2.4.1.217)/phosphatase (EC 3.1.3.70) was characterized previously from strain 195 (DET1363, *mgs*D) (Empadinhas *et al*., 2004). The two other genes were identified in *D* *. mccartyi* genomes during the metabolic modelling study (Ahsanul Islam *et al*., 2010).

The second purified enzyme (KB1_0553) was tested for both PGI/PMI activities using four standard assays (Experimental procedures). Although the PGI activity (Glucose‐6‐phosphate, G6P ↔ Fructose‐6‐phosphate, F6P) was tested in both directions with two assays, the enzyme showed no activity with either G6P or F6P as substrates (data not shown). The presence of PMI activity (Mannose‐6‐phosphate, M6P ↔ Fructose‐6‐phosphate, F6P) was confirmed in the direction of F6P formation using two assays (Experimental procedures). The pH range and kinetic parameters (Table 1) were also estimated using M6P as substrate. Thus, only the PMI activity was detected and confirmed for KB1_0553 (*Dm*PMI). This activity is typically associated with peptidoglycan and teichoic acid biosynthesis (Kanehisa *et al*., 2011). Therefore, the physiological role of *Dm*PMI in *D. mccartyi* is unclear because they lack a typical bacterial cell wall in favour of an archaeal S‐layer like protein and cell membrane (Löffler *et al*., 2012). *Dm*PMI may be involved in cell membrane biogenesis in these bacteria as suggested by the previous transcriptomic study (Ahsanul Islam *et al*., 2014). It can also be involved in the mannosylglycerate (MG) biosynthesis pathway (Fig. 1) because a bifunctional mannosyl‐3‐phosphoglycerate synthase (EC 2.4.1.217)/phosphatase (EC 3.1.3.70) enzyme encoded by DET1363 (*mgs*D) gene was biochemically characterized from *D. mccartyi* strain 195 (formerly *D. ethenogenes* strain 195) (Empadinhas *et al*., 2004). MG is an unusual compatible solute that helps thermophilic or hyperthermophilic bacteria and archaea to adjust osmotic stress and heat (Santos and da Costa, 2002; Empadinhas and da Costa, 2011). Because *D. mccartyi* strains are halotolerant or slightly halophilic (Empadinhas *et al*., 2004), MG was hypothesized to be involved in cellular osmotic stress adaptation in these bacteria (Hendrickson *et al*., 2002; Empadinhas *et al*., 2004). However, this suggested physiological role is a non‐essential or specialized function rather than a major biological function, which probably explains why *Dm*PMI has relatively lower catalytic activity and efficiency (Table 1) than similar enzymes in BRENDA (Chang *et al*., 2009).

### Sequence homology and phylogenetic analyses of *Dm*IDH and *Dm*PMI sequences

**Figure 2:**
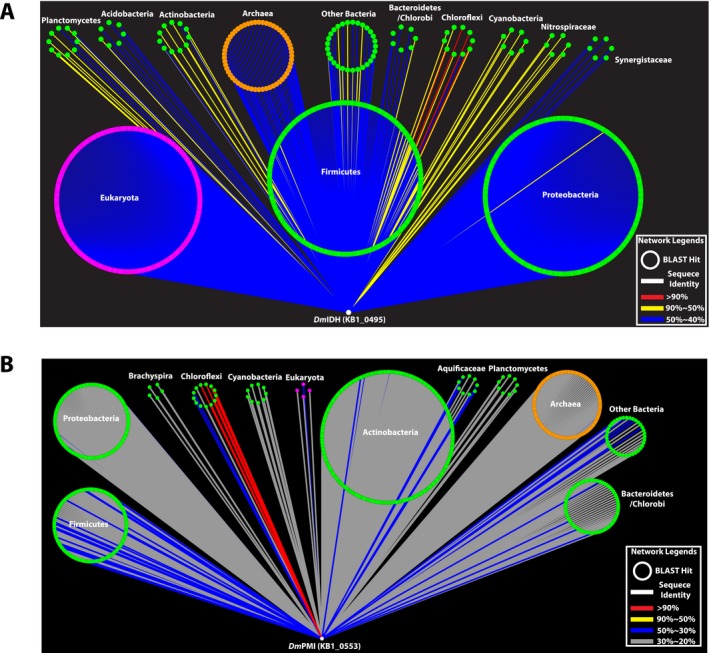
Sequence homology network of *Dm*IDH and *Dm*PMI. Homologous protein sequences of *Dm*IDH and *Dm*PMI in archaea, bacteria, and eukarya, identified by blastp in BLAST (Altschul *et al*., 1997) from UniProt (Apweiler *et al*., 2012), are shown as nodes, and sequence identities of *Dm*IDH and *Dm*PMI homologs are shown as edges in (A) and (B) respectively. The majority of *Dm*IDH homologs were > 40% identical at the amino acid level (indicated by blue edges), whereas the majority of *Dm*PMI homologs shared < 30% amino acid sequence identity with the *Dm*PMI sequence (indicated by grey edges).

**Figure 3:**
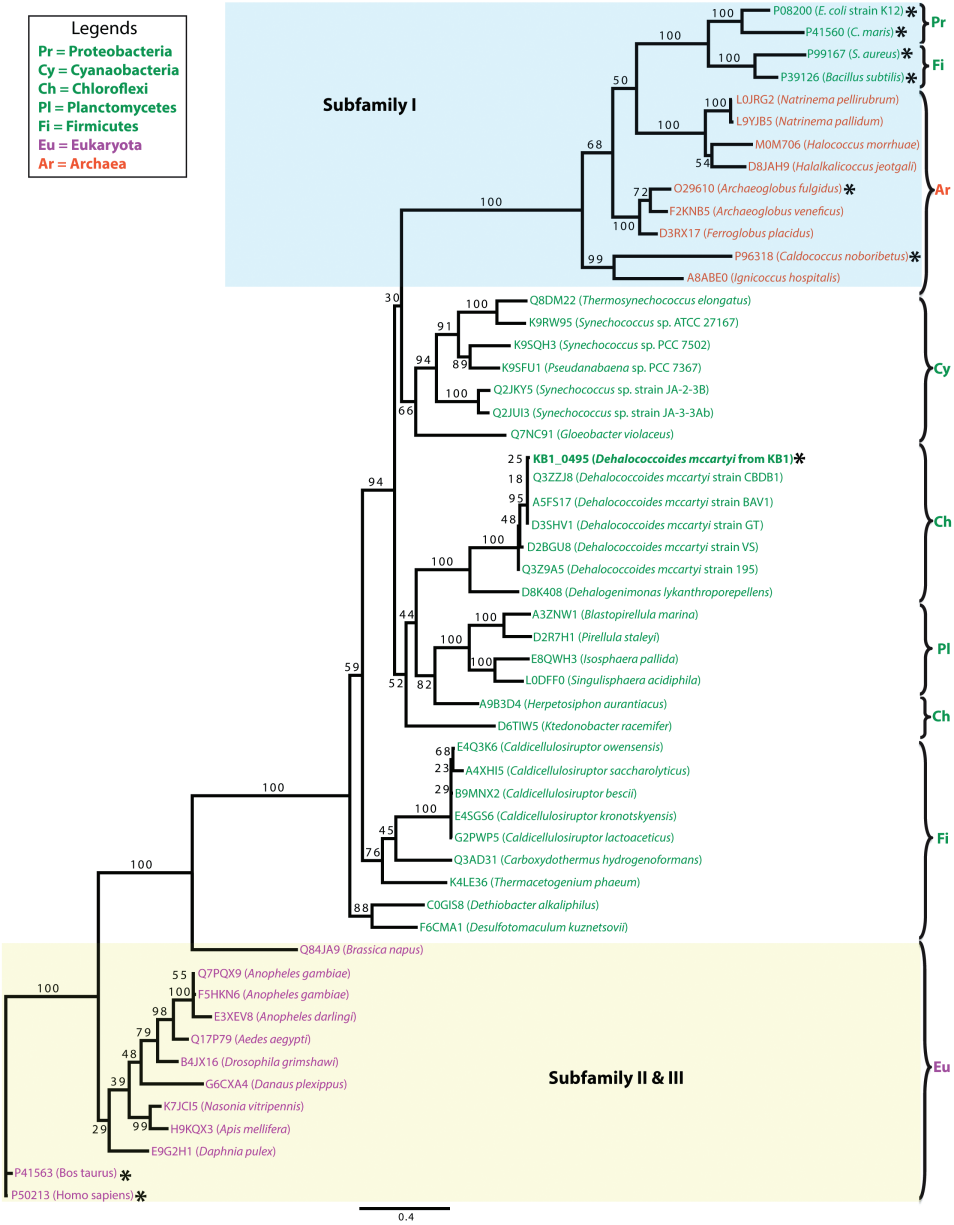
Phylogenetic analysis of *Dm*IDH protein sequence. Maximum likelihood (ML) tree for *Dm*IDH and its homologous protein sequences was constructed by P hy ML (Guindon *et al*., 2010) plugin in geneious (Biomatters, 2011). Protein sequences were mined from UniProt (Apweiler *et al*., 2012) and aligned with muscle (Edgar, 2004) plugin in geneious. Then, the ML tree was constructed under wag (Whelan and Goldman, 2001) model of amino acid substitution with 100 bootstrap resampling trees were conducted. Bootstrap values are shown as branch labels, and the biochemically characterized genes are marked by asterisks. Organism names are coloured according to different kingdoms (Orange = *Archaea*, Green = *Bacteria*, and Purple = *Eukarya*).

**Figure 4:**
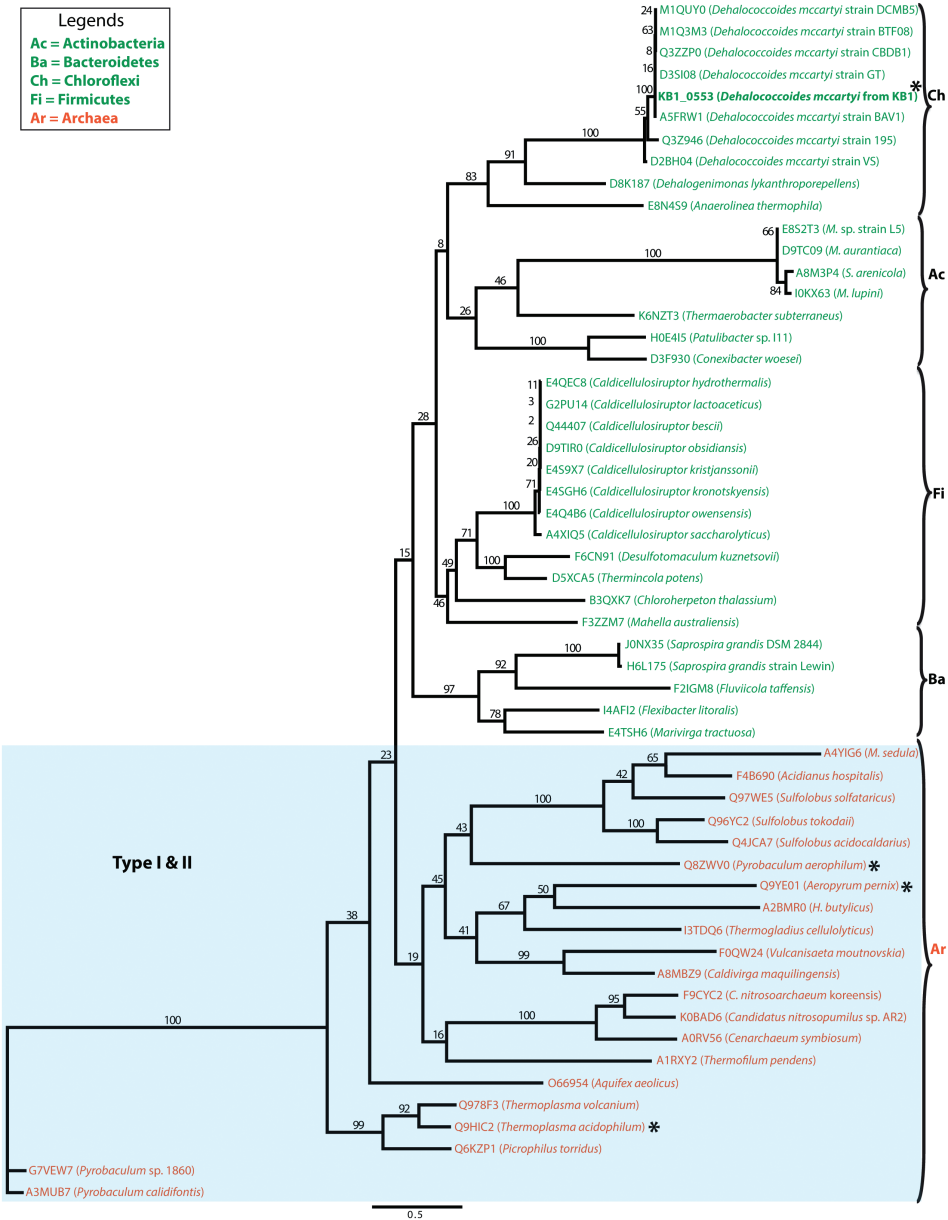
Phylogenetic analysis of *Dm*PMI protein sequence. Maximum likelihood (ML) tree for *Dm*PMI and its homologous protein sequences was constructed by P hy ML (Guindon *et al*., 2010) plugin in geneious (Biomatters, 2011). Protein sequences were mined from UniProt (Apweiler *et al*., 2012) and aligned with muscle (Edgar, 2004) plugin in geneious. Then, the ML tree was constructed under wag (Whelan and Goldman, 2001) model of amino acid substitution with 100 bootstrap resampling trees were conducted. Bootstrap values are shown as branch labels, and the biochemically characterized genes are marked by asterisks. Organism names are coloured according to different kingdoms (Orange = *Archaea* and Green = *Bacteria*).

The sequence homology analysis of *Dm*IDH and *Dm*PMI protein sequences (Fig. 2) revealed the remarkably conserved nature of *Dm*IDH across the domains of life (Fig. 2A) as compared with *Dm*PMI (Fig. 2B). Being a TCA‐cycle enzyme, *Dm*IDH was found to share > 40% amino acid sequence identity with other homologous IDHs from eukarya, archaea, and bacteria (Fig. 2A), whereas *Dm*PMI showed < 30% amino acid sequence identity with the majority of its homologs (Fig. 2B). This difference in sequence conservation was also observed from the higher bootstrap values in the *Dm*IDH maximum likelihood (ML) tree (Fig. 3) as compared with those in the ML tree of *Dm*PMI (Fig. 4). The *Dm*IDH tree further showed its separate clustering (Fig. 3) from the previously described (Steen *et al*., 1997) subfamilies I and II of biochemically characterized IDHs, as well as from subfamily III of NAD^+^‐dependent eukaryotic IDHs (Steen *et al*., 1997). Thus, *Dm*IDH belongs to a potentially new subfamily that also includes bacterial IDHs from *Planctomycetes*, *Firmicutes* and *Cyanobacteria* (Fig. 3). Interestingly, no homolog of *Dm*PMI was identified within the three types of PMIs characterized and described previously (Schmidt *et al*., 1992; Proudfoot *et al*., 1994). Among the biochemically characterized enzymes, the closest homologs of *Dm*PMI were identified to be archaeal bifunctional PGI/PMIs (Fig. 4), which represent a novel enzyme family within the PGI superfamily (Hansen *et al*., 2004a, 2004b). Thus, *Dm*PMI likely constitutes a novel class of bacterial PMIs, including the homologs from *Actinobacteria*, *Firmicutes* and *Bacteroidetes* (Fig. 4).

### Structure‐based analysis of *Dm*IDH and *Dm*PMI sequences

**Figure 5:**
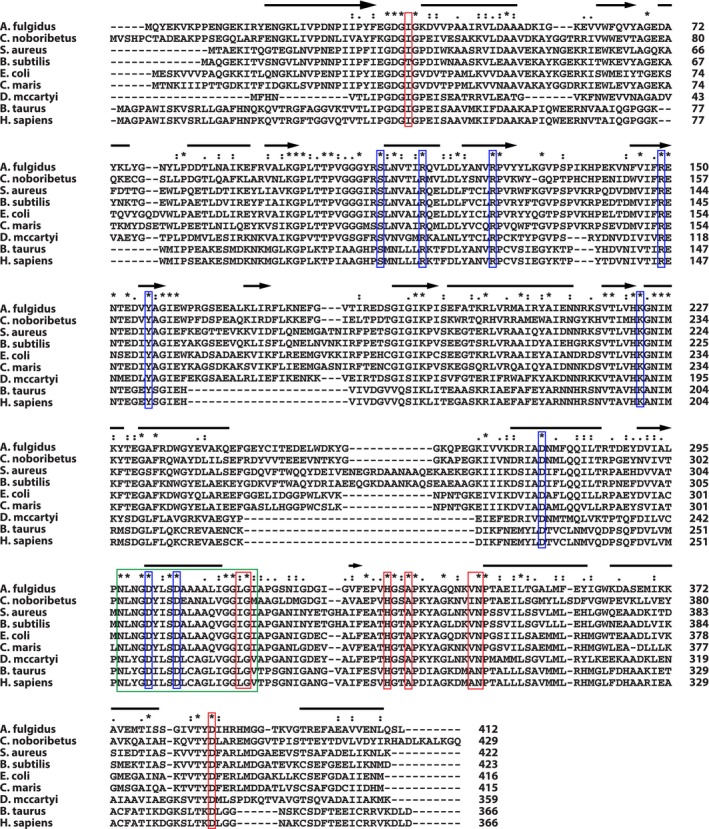
Structure‐based multiple sequence alignment (MSA) of *Dm*IDH. MSA of *Dm*IDH protein sequence and its homologous, biochemically characterized sequences are shown. Protein sequences were mined from the swissprot curated database (Boeckmann *et al*., 2003), and MSA was performed by C lustal X (Larkin *et al*., 2007). Completely conserved residues are marked by asterisks, whereas residues reported to be important for substrate and coenzyme binding (Hurley *et al*., 1991; Stokke *et al*., 2007) are marked by blue and red boxes respectively. Green box indicates signature motif for isocitrate/isopropylmalate dehydrogenases as identified by ScanProsite (de Castro *et al*., 2006). Protein secondary structure was predicted by PredictProtein (Rost and Sander, 1994), and indicated by bars (α‐helices) and arrows (β‐strands). Protein accession numbers (UniProt) are: *A* *. fulgidus* (O29610), *C* *. noboribetus* (P96318), *S* *. aureus* (P99167), *B* *. subtilis* (P39126), *E* *. coli* (P08200), *C* *. maris* (P41560), *B* *. taurus* (P41563) and *H* *. sapiens* (P50213).

To verify the aforementioned differences of *Dm*IDH and *Dm*PMI from the previously characterized similar enzymes, we conducted further bioinformatics analyses of their sequences. Multiple sequence alignment (MSA) of *Dm*IDH sequence and its biochemically characterized homologs from SWISSPROT identified 66 completely conserved residues (Fig. 5). These included all residues (indicated by blue and red boxes in Fig. 5) involved in substrate and coenzyme binding in *Archaeoglobus fulgidus* and *E. coli* IDHs (Hurley *et al*., 1991; Steen *et al*., 1997; Stokke *et al*., 2007), suggesting a similar reaction mechanism for *Dm*IDH. Although the signature motif of both isocitrate and isopropylmalate dehydrogenases (IDH and IPMDH) was identified in *Dm*IDH (indicated by a green box in Fig. 5), only the IDH activity was tested and confirmed because of its location in an operon containing other putative TCA‐cycle genes (Fig. S2). Also, the *D. mccartyi* genomes harbor a putative IPMDH gene (KB1_0839, cbdbA804, DET0826, DhcVS_730, DehaBAV1_0745 and DehalGT_0706) located in the L‐leucine biosynthesis operon (Fig. S4). Comparison of the predicted secondary structure of *Dm*IDH (Fig. 5) with the crystal structures of *A. fulgidus* and *E. coli* IDHs (Hurley *et al*., 1991; Stokke *et al*., 2007) showed that most helix and strand regions were conserved among them in spite of the presence of some subtle structural differences. For instance, *Dm*IDH has 12 α‐helices and 11 β‐strands (Fig. 5), whereas *A. fulgidus* has 18 α‐helices, 16 β‐strands (Stokke *et al*., 2007) and *E. coli* has 13 α‐helices, 12 β‐strands (Hurley *et al*., 1991).

**Figure 6:**
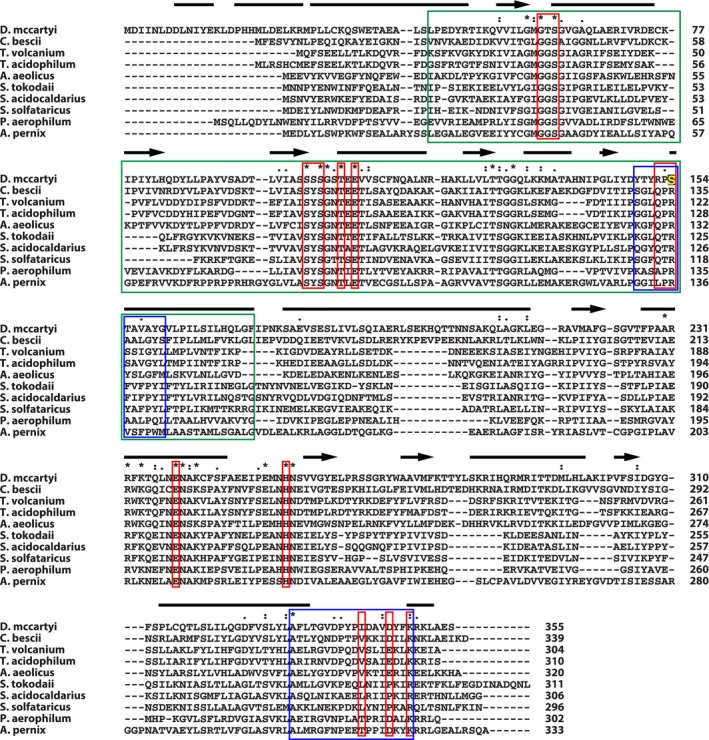
Structure‐based multiple sequence alignment (MSA) of *Dm*PMI. MSA of *Dm*PMI protein sequence and its homologous, manually reviewed/biochemically characterized sequences are shown. Protein sequences were mined from the swissprot curated database (Boeckmann *et al*., 2003), and MSA was performed by C lustal X (Larkin *et al*., 2007). Completely conserved residues are marked by asterisks, whereas residues reported to be important for substrate binding and catalysis (Hansen *et al*., 2004a; Swan *et al*., 2004) are marked by red boxes. Blue boxes indicate two signature motifs for bifunctional PGI/PMI protein family (Hansen *et al*., 2004b), and green box indicates Pfam (Punta *et al*., 2012) SIS (sugar isomerase) domain identified by ScanProsite (de Castro *et al*., 2006). Protein secondary structure was predicted by P redict P rotein (Rost and Sander, 1994), and indicated by bars (α‐helices) and arrows (β‐strands). Protein accession numbers (UniProt) are: *C* *. bescii* (Q44407), *T* *. volcanium* (Q978F3), *T* *. acidophilum* (Q9HIC2), *A* *. aeolicus* (O66954), *S* *. tokodaii* (Q96YC2), *S* *. acidocaldarius* (Q4JCA7), *S* *. solfataricus* (Q97WE5), *P* *. aerophilum* (Q8ZWV0) and *A* *. pernix* (Q9YE01).

MSA of *Dm*PMI sequence with its biochemically characterized or manually reviewed homologs identified only 20 completely conserved residues (Fig. 6). In addition to two signature motifs (marked by blue boxes in Fig. 6) of archaeal PGI/PMIs (Hansen *et al*., 2004b), a SIS (sugar isomerase) domain (marked by a green box in Fig. 6) was identified in *Dm*PMI. All residues proposed to be important for substrate binding and catalysis in archaeal PGI/PMIs (Hansen *et al*., 2004a; Swan *et al*., 2004) are also found to be conserved in *Dm*PMI (marked by red boxes in Fig. 6), except a few notable ones: Thr60, Ser103, Arg152, Ser154, Pro341, and Ile342. The most important change was detected at residue position 154, where a Serine (S) substituted an Arginine (R) found in other archaeal PGI/PMIs (marked by a yellow S in Fig. 6). This residue is pivotal for the catalytic activities of archaeal PGI/PMIs because it stabilizes the formation of crucial enediol intermediates during their PGI activity (Seeholzer, 1993; Hansen *et al*., 2004b; Swan *et al*., 2004). Hence, these changes in the *Dm*PMI sequence are likely responsible for its inability to function as PGI. A similar notion was also obtained from the comparison of the predicted secondary structure of *Dm*PMI (12 α–helices and 9 β‐strands) (Fig. 6) with the crystal structure of *Pa*PGI/PMI from *P. aerophilum* (Swan *et al*., 2004). However, the mechanism for PMI activity of *Dm*PMI is likely similar to *Pa*PGI/PMI because the presence of Thr291 in the *Pa*PGI/PMI sequence is key to its PMI activity (Swan *et al*., 2004), and the equivalent residue in *Dm*PMI is an Isoleucine (Ile342) (Fig. 6).

In summary, the two characterized enzymes, *Dm*IDH and *Dm*PMI, from *D. mccartyi* strains in KB‐1 likely have reaction mechanisms similar to the previously characterized enzymes although they appear to belong to novel IDH and PMI enzyme families. *Dm*IDH showed a higher catalytic activity with NADP^+^ as a cofactor than with NAD^+^, implicating its involvement in anabolic biosynthetic pathways in *D. mccartyi*, whereas the lower activity of *Dm*PMI suggests that the physiological substrate is possibly a different sugar than M6P, or that it is possibly involved in non‐essential physiological roles such as osmotic stress adjustment in these bacteria.

## Conclusions

Automated and non‐curated primary annotations of genes are necessary but highly problematic if they are wrong. Because initial annotations are often non‐specific or incorrect, correcting these annotations is very important and challenging. In this study, corrected or reviewed initial annotations that were proposed from previously published modelling and transcriptomic studies were experimentally tested and shown to be essentially correct, illustrating the utility of metabolic modelling and bioinformatics approaches as important tools of hypothesis generation for gene annotations. Each new biochemical confirmation of gene functions adds to the confidence of propagated annotations, contributing to a stronger database for sequence analysis in general. In the specific case of the unusual organohalide respiring anaerobes *D. mccartyi*, fundamental understanding of the physiology and biochemistry of these organisms will contribute to better deployment of bioremediation approaches for environmental stewardship.

## Experimental procedures

### Bacterial culture, reagents, and chemicals

Genomic DNA (gDNA) was collected from KB‐1, a *D. mccartyi*‐containing anaerobic mixed culture growing on TCE and methanol following the procedure described previously (Duhamel and Edwards, 2006). The PCR primers for amplifying *D. mccartyi* gDNA were synthesized by Integrated DNA Technologies (Coralville, IA, USA). Luria broth and terrific broth powder were purchased from EMD Chemicals (Gibbstown, NJ, USA), and the Bradford assay reagent from Bio‐Rad (Hercules, CA, USA). Lysozyme, proteinase K, agarose, glycerol, ampicillin, kanamycin, SDS and IPTG were obtained from BioShop (Burlington, ON, Canada), and all other chemicals were purchased from Sigma‐Aldrich (St. Louis, MO, USA) with greater than 98% in purity. Nickel‐Nitrilotriacetic acid (Ni‐NTA) resin and the QIAquick PCR Purification Kit were purchased from Qiagen (Mississauga, ON, Canada), whereas the In‐Fusion PCR Cloning Kit was purchased from Clontech (Palo Alto, CA, USA). The commercially available kits were used according to the manufacturers' instructions.

### Gene cloning, protein overexpression, and purification

The selected genes (KB1_0495 and KB1_0553) were PCR‐amplified using *D. mccartyi* gDNA and the PCR primers containing the restriction sites for BamHI and NdeI, and were cloned into the modified pET‐15b vector (Novagen, Madison, WI, USA) containing a 5′ N‐terminal hexahistidine tag (6xHis‐tag) and an ampicillin resistance gene as described previously (Zhang *et al*., 2001). In the modified vector, the tobacco etch virus protease cleavage site replaced the thrombin cleavage site, and a double stop codon was introduced downstream from the BamHI site (Zhang *et al*., 2001). These vectors were subsequently transformed into *E. coli* strain BL21 (DE3) (Stratagene, La Jolla, CA, USA) for overexpression of the targeted fused genes. The cells were grown aerobically in 1 L flasks containing tryptone‐phosphate medium at 37°C and ∼ 220 rpm until the OD_600_ reached around 1.0 (approximately in 3 h). Expression of the cloned genes was induced by adding 100 mg IPTG, and cells were harvested the following day by centrifugation (Zhang *et al*., 2001). The overexpressed, fused 6xHis‐tagged proteins were purified to more than 95% homogeneity (Fig. S1) using metal‐chelate affinity chromatography on nickel affinity resin and gel filtration on a Superdex 200 26/60 column (Amersham Biosciences, Piscataway, NJ, USA) as described before (Zhang *et al*., 2001; Proudfoot *et al*., 2008).

### Isocitrate dehydrogenase (IDH) assay

IDH activity in KB1_0495 (*Dm*IDH) was confirmed by an enzymatic assay (Steen *et al*., 1997; 1998) for the reaction described by the following equation:D−isocitric acid+NAD(P)+ ⇔IDH 2−oxoglutarate + CO2+NAD(P)H


The standard 1 ml assay contained 50 mM tris‐hydrochloride (Tris‐HCl) buffer (pH 7.5), 0.3 mM NADP^+^, 1 mM D‐isocitric acid and 10 mM MgCl_2_. The reaction was started by adding 1 μg of purified protein into the reaction mixture, and the product formation was inferred from the measurement of NAD(P)H formation as indicated by the increase in absorbance at 340 nm and 30°C with a spectrophotometer. The IDH activity in KB1_0495 was also measured using NAD^+^ as a cofactor.

### Phosphoglucose isomerase (PGI) assay

PGI activity in KB1_0553 (*Dm*PMI) was tested using two standard assays (Hansen *et al*., 2004b) for the reaction described by the following equations:

Assay 1:$F6P\;\overset{PGI}{\Leftrightarrow }\;G6P$
$G6P+NADP^{+}\;\overset{G6PDH}{\Leftrightarrow }\;6PG15L+NADPH$where F6P refers to fructose‐6‐phosphate, G6P refers to glucose‐6‐phosphate, 6PG15L refers to 6‐phospho‐D‐glucono‐1,5‐lactone, and G6PDH is glucose‐6‐phosphate dehydrogenase (EC. 1.1.1.49).

Assay 2:$G6P\;\overset{PGI}{\Leftrightarrow }\;F6P$
$F6P+NADH\;\overset{M1PDH}{\Leftrightarrow }\;M1P+NAD^{+}$where M1P refers to mannitol‐1‐phosphate and M1PDH is mannitol‐1‐phosphate‐5‐dehydrogenase (EC 1.1.1.17). Reaction mixtures for assay 1 contained 100 mM Tris‐HCl (pH 7.5), 0.5 mM NADP^+^, 10 mM F6P, and 1.1 U of G6PDH (Sigma‐Aldrich, St. Louis, MO), and assay 2 contained 100 mM Tris‐HCl (pH 7.5), 0.3 mM NADH, 10 mM G6P, and 10 μL of M1PDH. The M1PDH was purified from *E. coli* (Novotny *et al*., 1984) with > 95% purity (see Fig. S1). The reactions were started by adding 1 μg of purified protein into the reaction mixture, and the product formation was inferred from the measurement of NADPH formation in assay 1 and of oxidation of NADH in assay 2 with a spectrophotometer at 340 nm absorbance and 30°C.

### Phosphomannose isomerase (PMI) assay

PMI activity in KB1_0553 (*Dm*PMI) was tested with two standard assays (Hansen *et al*., 2004a) for the reactions described by the following equations:

Assay 1:$M6P\;\overset{PMI}{\Leftrightarrow }\;F6P$
$F6P+NADH\;\overset{M1PDH}{\Leftrightarrow }\;M1P+NAD^{+}$where M6P refers to mannose‐6‐phosphate.

Assay 2:$M6P\;\overset{PMI}{\Leftrightarrow }\;F6P$
$F6P\;\overset{PGI}{\Leftrightarrow }\;G6P$
$G6P+NADP^{+}\;\overset{G6PDH}{\Leftrightarrow }\;6PG15L+NADPH$


The reaction mixture for assay 1 contained 100 mM Tris‐HCl (pH 7.5), 0.5 mM NADH, 10 mM M6P, and 10 μl of M1PDH. The M1PDH was purified from *E. coli* (Novotny *et al*., 1984) with > 95% purity (see Fig. S1). In assay 2, the reaction mixture contained 100 mM Tris‐HCl (pH 7.5), 0.5 mM NADP^+^, 10 mM M6P and 1.1 U of G6PDH (Sigma‐Aldrich, St. Louis, MO) and 1 U of PGI (Sigma‐Aldrich, St. Louis, MO). The reactions were started by adding 1 μg of purified protein into the reaction mixture, and the product, F6P formation, was measured by coupling it to the oxidation of NADH in assay 1 and to the formation of NADPH in assay 2. In both instances, the product formation was inferred from the measurement of absorbance with a spectrophotometer at 340 nm and 30°C.

### Determination of pH range and kinetic parameters

The pH range of purified KB1_0495 (*Dm*IDH) and KB1_0553 (*Dm*PMI) (Table 1) was determined by measuring their activities at pH values from 6 to 8.5 at 30°C using D‐isocitric acid and M6P as substrates, respectively. The enzymatic activity of both purified proteins was also measured at selected pHs with varying concentrations of substrates, D‐isocitric acid, M6P and cofactors, NADP^+^ and NAD^+^ at 30°C. These data were, then, fitted to the Michaelis–Menten enzyme kinetics model with the non‐linear regression analysis to estimate the maximum enzyme velocity (*V*_max_) and the Michaelis constant (*K*_m_) using GraphPad Prism v 5.0 (GraphPad Software, La Jolla, CA, USA). Then, the turnover number (*k*_cat_) and the efficiency (*k*_cat_/*K*_m_) of both proteins were calculated from *V*_max_ and *K*_m_ data.

### Bioinformatics analyses of *Dm*IDH and *Dm*PMI sequences

Homologous protein sequences of *Dm*IDH and *Dm*PMI in eukarya, archaea, and bacteria were identified by blastp in blast (Altschul *et al*., 1997) from the UniProt database (Apweiler *et al*., 2012). The sequence homology networks of both *Dm*IDH and *Dm*PMI were constructed and visualized by cytoscape (Smoot *et al*., 2011) – an open source bioinformatics software platform. The phylogenetic analysis was conducted by the PhyML (Guindon *et al*., 2010) plugin in the geneious software platform (Biomatters, 2011) to construct the ML trees for both *Dm*IDH and *Dm*PMI sequences and their homologous protein sequences. First, the homologous sequences were mined from UniProt (Apweiler *et al*., 2012) with blastp in blast and aligned with the muscle (Edgar, 2004) plugin in geneious. Then, the ML tree was constructed using the wag (Whelan and Goldman, 2001) model of amino acid substitution with 100 bootstrap resampling trees were conducted. The multiple sequence alignment of *Dm*IDH and *Dm*PMI protein sequences was performed by ClustalX (Larkin *et al*., 2007), and the biochemically characterized homologous protein sequences were mined from the SWISSPROT curated database (Boeckmann *et al*., 2003). The protein secondary structure of *Dm*IDH and *Dm*PMI was predicted by the PredictProtein (Rost and Sander, 1994) software.

## Conflict of interest

None declared.

## Supporting information


**Fig. S1.** SDS‐PAGE of (A) *Dm*IDH, (B) *Dm*PMI, and (C) M1PDH.
**Fig. S2.** Orthologous gene neighborhood analysis of isocitrate dehydrogenase (IDH) from *D. mccartyi*.
**Fig. S3.** Orthologous gene neighborhood analysis of hypothetical protein/SIS domain protein from *D. mccartyi*.
**Fig. S4.** Orthologous gene neighborhood analysis of 3‐isopropylmalate dehydrogenase (IPMDH) from *D. mccartyi*
