# Supplementary material for: Experimental validation of in silico model‐predicted isocitrate dehydrogenase and phosphomannose isomerase from D ehalococcoides mccartyi
Source: Microb Biotechnol. 2015 Sep 16;9(1):47–60. doi: 10.1111/1751-7915.12315 (PMC4720418; doi:10.1111/1751-7915.12315)
Supplement: Supplementary file 1 — Fig. S1. SDS‐PAGE of (A) DmIDH, (B) DmPMI, and (C) M1PDH. Fig. S2. Orthologous gene neighborhood analysis of isocitrate dehydrogenase (IDH) from D. mccartyi. Fig. S3. Orthologous gene neighborhood analysis of hypothetical protein/SIS domain protein from D. mccartyi. Fig. S4. Orthologous gene neighborhood analysis of 3‐isopropylmalate dehydrogenase (IPMDH) from D. mccartyi [file MBT2-9-047-s001.pdf]

Supplementary information

Experimental Validation of *In Silico* Model-Predicted Isocitrate dehydrogenase and Phosphomannose isomerase from *Dehalococcoides mccartyi*

M. Ahsanul Islam*, Anatoli Tchigvintsev, Veronica Yim, Alexei Savchenko, Alexander F. Yakunin, Radhakrishnan Mahadevan, and Elizabeth A. Edwards#

Department of Chemical Engineering and Applied Chemistry, University of Toronto, Ontario, Canada

Running Head: Functional characterization of *D. mccartyi* proteins

#Address correspondence to elizabeth.edwards@utoronto.ca

*Present address: Department of Chemical Engineering, Massachusetts Institute of Technology, Cambridge, Massachusetts, USA

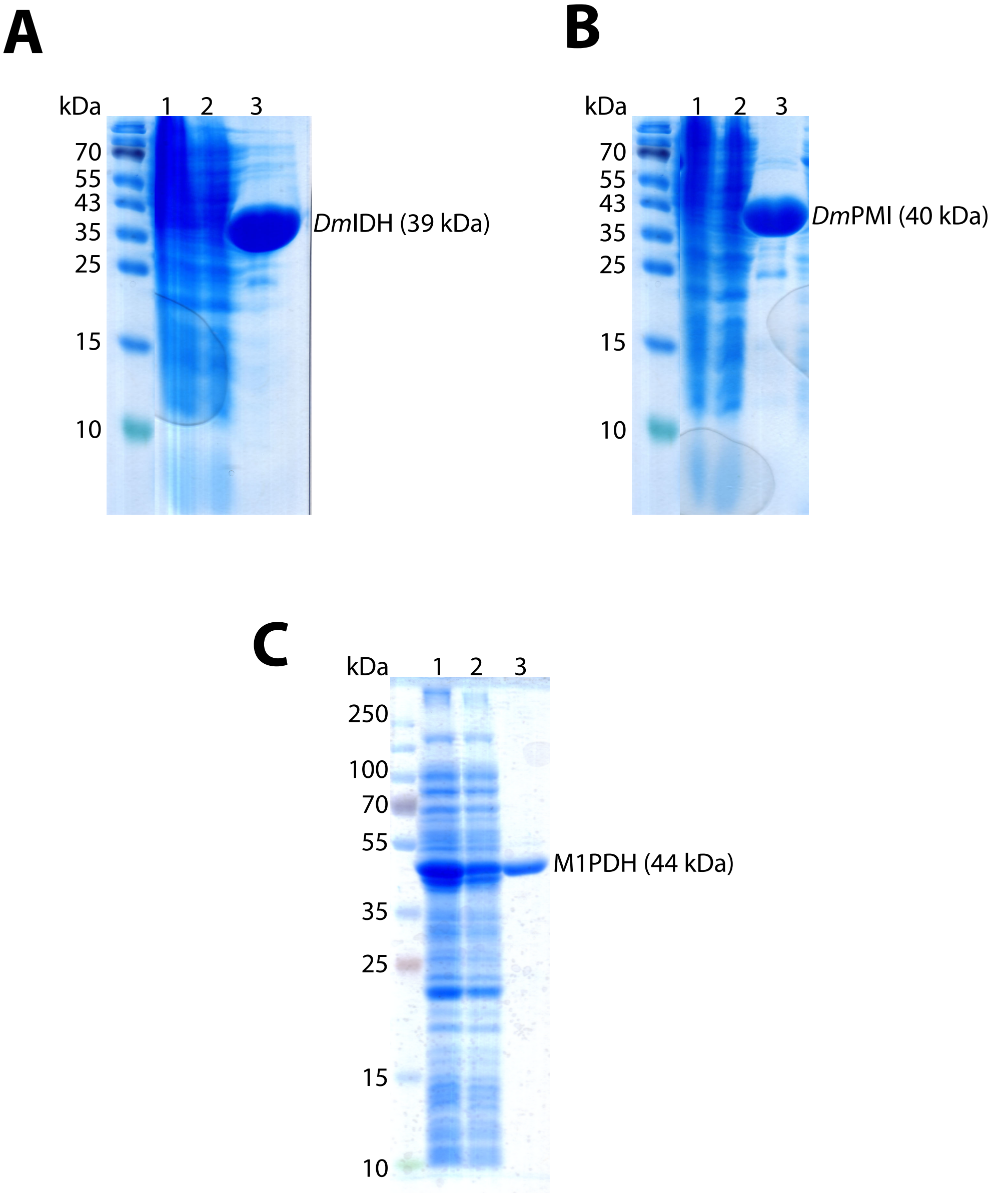

Figure S1. SDS-PAGE of (A) *Dm*IDH, (B) *Dm*PMI, and (C) M1PDH. In each case, protein standard is given on the left, and the gene product is shown on the right. Also, the protein fractions in each case are: (1) crude extract (supernatant), (2) flow-through from a Ni-column purification unit, (3) eluted proteins.

Selection of KB1_0495 (*Dm*IDH) and KB1_0553 (*Dm*PMI) for experimental verification

The gene encoding a putative isocitrate dehydrogenase (IDH) enzyme in *D. mccartyi* genomes (DET0450, cbdbA408, DehaBAV_0427, DhcVS_392, DehalGT_0391, btf_415, dcmb_461, GY50_0375, and KB1_0495) was annotated as an NAD^+-dependent isocitrate dehydrogenase (EC. 1.1.1.41) (Kube, et al., 2005, Seshadri, et al., 2005, Markowitz, et al., 2012). The annotation of this gene is also not very specific in biological databases, including COG (Tatusov, et al., 2000), TIGR Pfam (Green and Klein, 2002), and EBI Pfam (Punta, et al., 2012), where it is listed as belonging to the isocitrate/isopropylmalate dehydrogenase protein family. However, during the construction and manual curation of the *D. mccartyi* metabolic model (Ahsanul Islam, et al., 2010), we reannotated this gene as an $NADP^+$-dependent IDH (EC. 1.1.1.42) based on bioinformatic analysis of the gene sequence. Also, the orthologous gene neighborhood analysis i.e., the analysis of the operon structure of a gene of interest in orthologous genomes (Markowitz, et al., 2012), revealed that the putative IDH gene is located in an operon with other TCA-cycle genes, including the large and small subunits of a putative aconitase, a putative malate dehydrogenase, and a putative fumarate hydratase (alpha and beta subunits) (Figure S2 in supplementary information). This operon information further corroborates its reannotation as an $NADP^+$-dependent IDH. However, due to the non-specific nature of annotation in biological databases and given the importance of the putative function of this gene in the TCA-cycle, we decided to experimentally characterize the putative IDH from *D. mccartyi* in KB-1 (KB1_0495).

The second gene (KB1_0553) was primarily annotated as a hypothetical protein/SIS domain protein in *D. mccartyi* genomes (DET0509, cbdbA472, DehaBAV1_0485, DhcVS_0450, DehalGT_0448, btf_472, dcmb_518, GY50_0435, and KB1_0553). Biological databases such as COG (Tatusov, et al., 2000) and EBI Pfam (Punta, et al., 2012) annotated the gene as a putative glucose-6-phosphate isomerase, while SEED (Overbeek, et al., 2005), TIGR Pfam (Green and Klein, 2002), and IMG (Markowitz, et al., 2012) annotated it as a bifunctional phosphoglucose isomerase (PGI; EC 5.3.1.9)/phosphomannose isomerase (PMI; EC 5.3.1.8). This gene was also identified to be a putative PGI/PMI during the construction and manual curation of the *D. mccartyi* metabolic model (Ahsanul Islam, et al., 2010); however, the annotation was given low confidence in the model because the gene sequence was < 30% identical at the amino acid level with other known homologous PGI/PMIs in biological databases. When we undertook a systems-level study of *D. mccartyi* transcriptomes (Ahsanul Islam, et al., 2014), we found that a homolog of KB1_0553 in *D. mccartyi* strain 195 genome (DET0590) was in a co-expressed gene-cluster enriched for central carbon metabolism genes involved in sugar metabolism. In addition, the orthologous gene neighborhood analysis (Markowitz, et al., 2012) revealed that the gene is located in an operon upstream of a putative bifunctional phosphoglucomutase/phosphomannomutase (Figure S3 in supplementary information), supporting its putative reannotation as a sugar metabolism gene. Thus, we decided to experimentally verify if KB1_0553 is actually a bifunctional PGI/PMI.

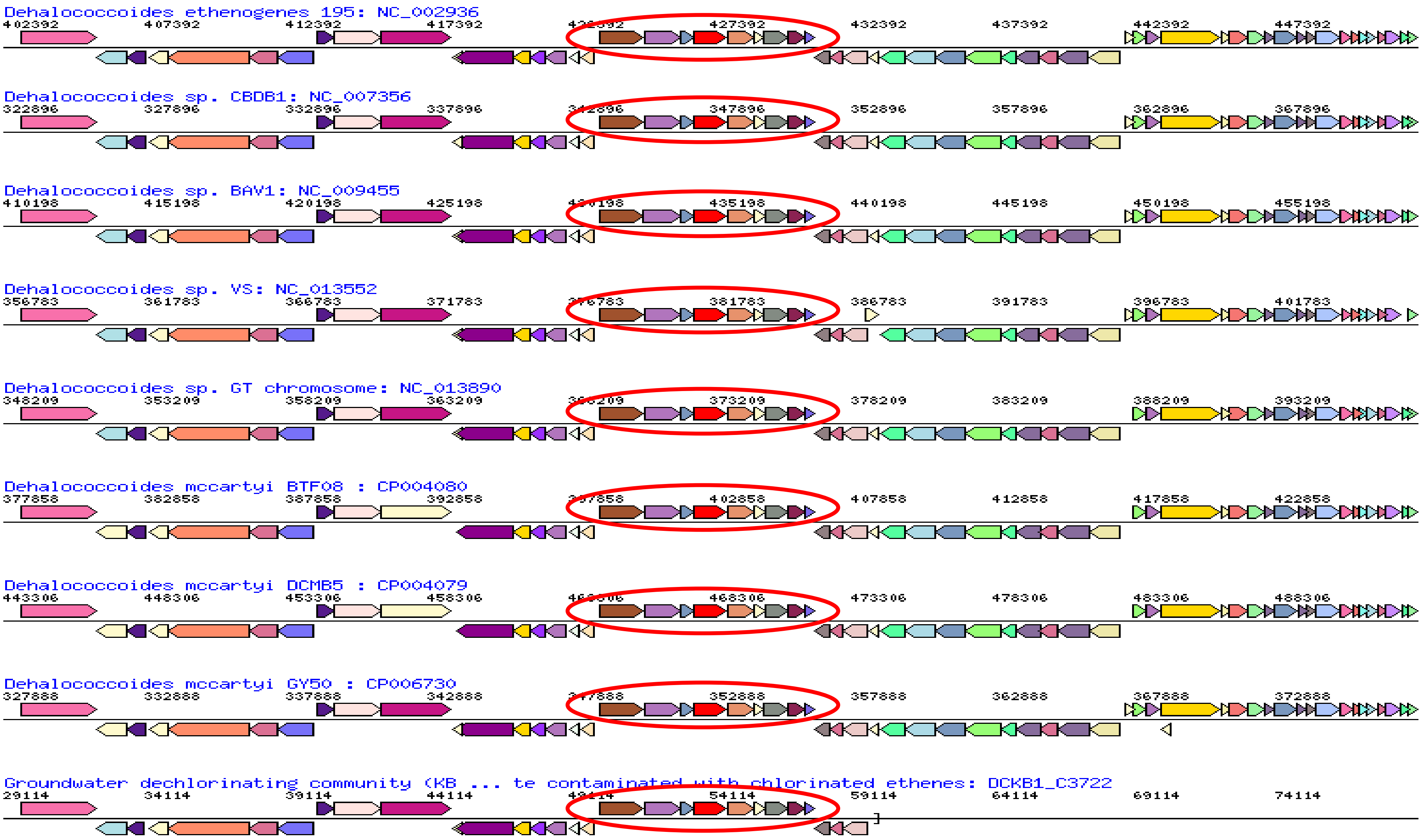

Figure S2. Orthologous gene neighborhood analysis of isocitrate dehydrogenase (IDH) from *D. mccartyi*. Genes that are orthologous to IDH in all *D. mccartyi* genomes (DET0450, cbdbA408, DehaBAV_0427, DhcVS_392, DehalGT_0391, btf_415, dcmb_461, GY50_0375, KB1_0495/DCKB1_131750) are shown by red color. Annotations of all genes in the operon (marked by a red circle) containing IDH are (from left to right): propionyl-CoA carboxylase, aconitase-large subunit, aconitase-small subunit, isocitrate dehydrogenase, malate dehydrogenase, hypothetical protein, fumarate hydratase-alpha subunit, fumarate hydratase-beta subunit, and HIT domain protein (1).

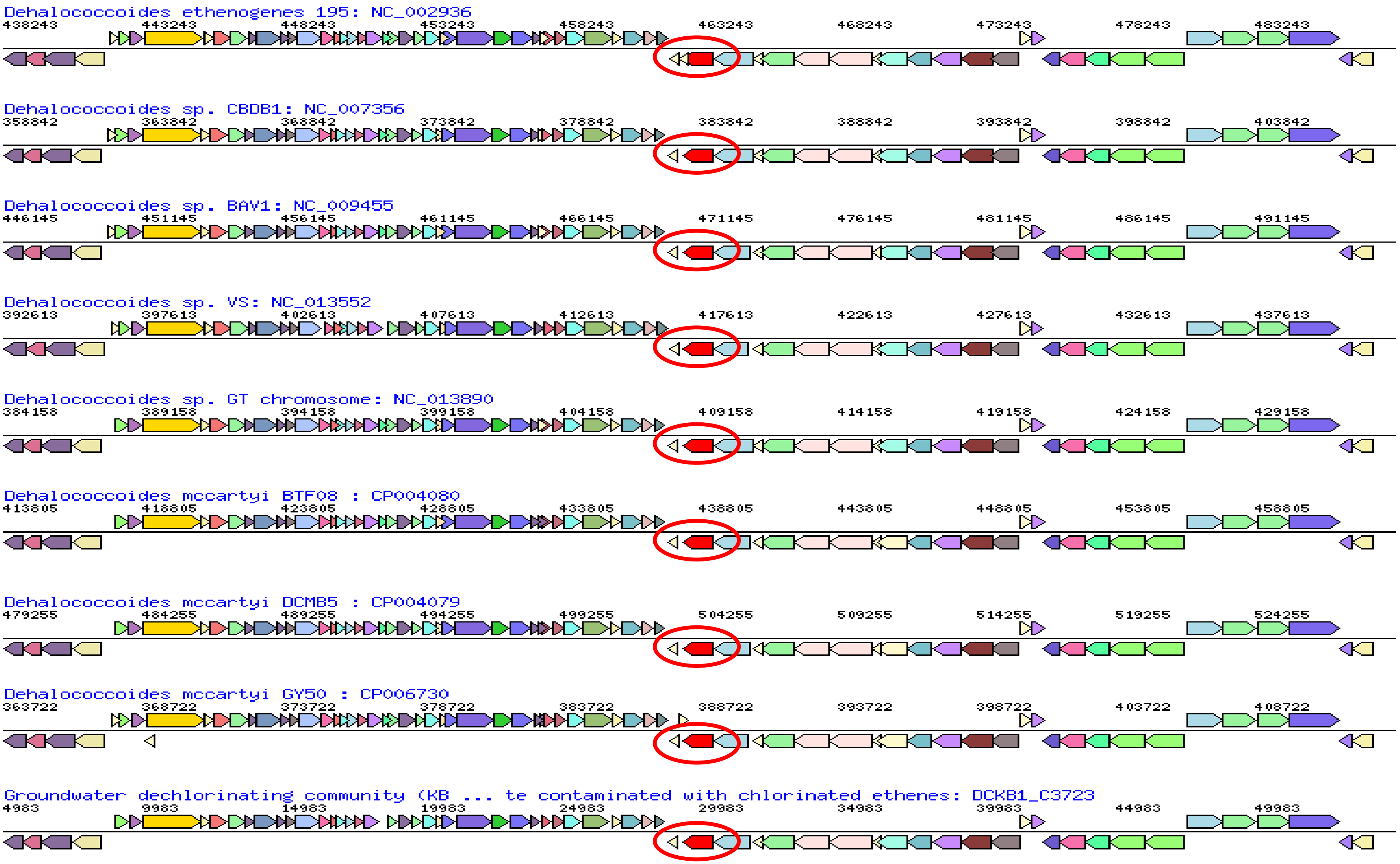

Figure S3. Orthologous gene neighborhood analysis of hypothetical protein/SIS domain protein from *D. mccartyi*. Genes that are orthologous to hypothetical protein/SIS domain protein in all *D. mccartyi* genomes (DET0509, cbdbA472, DehaBAV1_0485, DhcVS_0450, DehalGT_0448, btf_472, dcmb_518, GY50_0435, KB1_0553/DCKB1_132300) are shown by red color. The other gene in the operon (marked by a red circle) with hypothetical protein/SIS domain protein is a putative bifunctional phosphoglucomutase/phosphomannomutase (from left to right) (1).

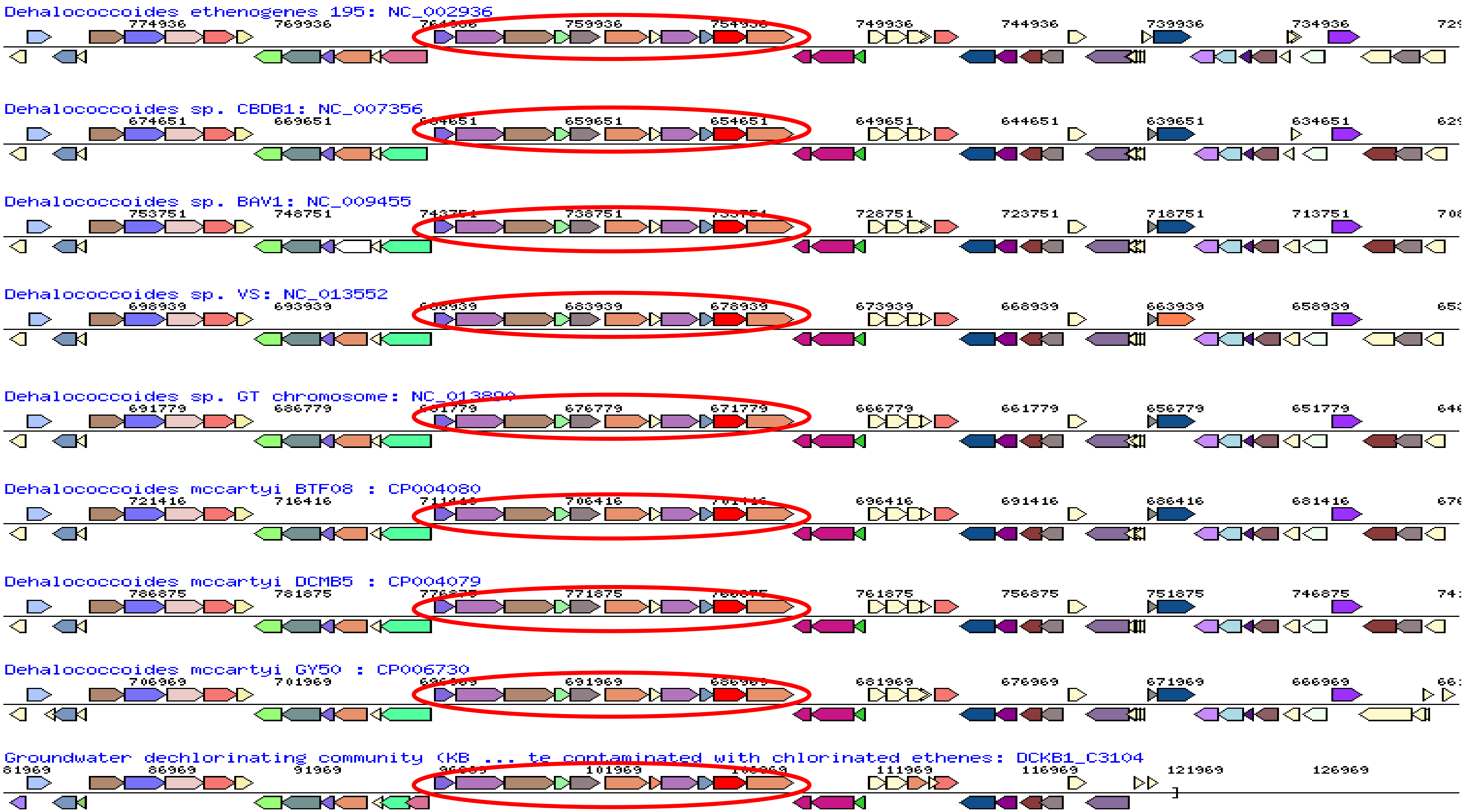

Figure S4. Orthologous gene neighborhood analysis of 3-isopropylmalate dehydrogenase (IPMDH) from *D. mccartyi*. Genes that are orthologous to IPMDH in all *D. mccartyi* genomes (DET0826, cbdbA804, DhcVS_730, DehaBAV1_0745, DehalGT_0706, btf_747, dcmb_793, GY50_0740, KB1_0839/DCKB1_106530) are shown by red color. Annotations of all genes in the operon (marked by a red circle) containing the IPMDH are (from left to right): putative translation factor, dihydroxy-acid dehydratase (ilvD), acetolactate synthase-large subunit (ilvB), acetolactate synthase-small subunit (ilvN), ketol-acid reductoisomerase (ilvC), 2-isopropylmalate synthase (leuA), membrane protein, 3-isopropylmalte dehydratase-large subunit (leuC), 3-isopropylmalate dehydratase-small subunit (leuD), 3-isopropylmalate dehydrogenase (leuB), 2-isopropylmalate synthase (1).

References

1. **Markowitz VM, Chen I, Palaniappan K, Chu K, Szeto E, Grechkin Y, Ratner A, Jacob B, Huang J, Williams P, Huntemann M, Anderson I, Mavromatis K, Ivanova N, Kyrpides N.** 2012. IMG: the Integrated Microbial Genomes database and comparative analysis system. Nucleic Acids Res. **40:**D115-122.
